# Plasticity of the Central Nervous System Involving Peripheral Nerve Transfer

**DOI:** 10.1155/2022/5345269

**Published:** 2022-03-18

**Authors:** Jun Shen

**Affiliations:** Department of Orthopaedic Oncology, The Second Affiliated Hospital of Naval Medical University, Naval Medical University, No. 415 Fengyang Road, Huangpu District, Shanghai, China

## Abstract

Peripheral nerve injury can lead to partial or complete loss of limb function, and nerve transfer is an effective surgical salvage for patients with these injuries. The inability of deprived cortical regions representing damaged nerves to overcome corresponding maladaptive plasticity after the reinnervation of muscle fibers and sensory receptors is thought to be correlated with lasting and unfavorable functional recovery. However, the concept of central nervous system plasticity is rarely elucidated in classical textbooks involving peripheral nerve injury, let alone peripheral nerve transfer. This article is aimed at providing a comprehensive understanding of central nervous system plasticity involving peripheral nerve injury by reviewing studies mainly in human or nonhuman primate and by highlighting the functional and structural modifications in the central nervous system after peripheral nerve transfer. Hopefully, it will help surgeons perform successful nerve transfer under the guidance of modern concepts in neuroplasticity.

## 1. Introduction

In clinical practice, peripheral nerve transfer (PNT), being an effective addition to medical interventions, has been commonly employed in patients with peripheral nerve injury or cervical spinal cord injury as a surgical salvage for restoration of the crucial function of paralyzed limbs [1–10]. Healthy donor nerves with less vital roles are sacrificed and transferred to the sites of damaged nerves [8]. After PNT, the axons of the donor nerves are expected to functionally reinnervate the formerly paralyzed muscles to regain favorable control of disabled limbs. However, ideal recovery of normal sensation and muscle control cannot be achieved even after complete nerve regeneration [11–13]. Surgeons are becoming increasingly aware of the alterations of the brain and spinal cord circuit triggered by peripheral nerve injury, which can exert a negative impact on the eventual functional restoration. These alterations have been termed neuroplasticity [14].

Plasticity is a unique biological property that refers to the ability of the central nervous system (CNS) to modify itself functionally and structurally in response to changes or demands within the organism itself and the external environment. This allows for the CNS to be constructed out of other body parts, such as the cardiovascular, respiratory, and digestive systems [15]. The most impressive form of neuroplasticity is the capacity for continuous neurogenesis during adulthood [16]. The history of neuroplasticity research involving peripheral nerve injury could date back as far as 1895 [17]. And it began with the phenomenon of “false localization,” which refers to the patient's inability to accurately localize a point of stimulation on the skin despite good regeneration of sensory fibers after median nerve transection [17, 18]. During the following century, a large number of animal experiments in nonhuman primates, cats, and raccoons were performed to examine the implicit functional and structural modifications in the CNS triggered by peripheral nerve injury [18–20]. In recent decades, well-developed recording technology has further boosted our knowledge of cortical and subcortical plasticity at the cellular and neuronal circuit levels in rodent models. Meanwhile, bold oxygen level-dependent functional magnetic resonance imaging (bold fMRI) developed in the 1990s has provided a profound understanding of this issue in humans. However, the concept of neuroplasticity has rarely been elucidated in classical textbooks involving peripheral nerve injury, let alone PNT. This article is aimed at providing a comprehensive understanding of the neuroplasticity involving peripheral nerve injury by reviewing studies mainly in human or nonhuman primates and by highlighting the functional and structural modifications in the CNS after PNT.

## 2. Plasticity of the CNS Involving Peripheral Nerve Injury

### 2.1. Time-Dependent Plasticity of the CNS after Sensory Deafferentation and Motor Deefferentation

The earliest experimental studies on peripheral nerve injury-related plasticity of the CNS were performed on animal models of mere sensory deafferentation, which included the transection of the median nerve at the wrist level (restricted deafferentation model) and resection of the dorsal roots of the spinal cord (extensive deafferentation model) [21, 22]. Plasticity of the CNS induced by sensory deafferentation is characterized by time-dependent features [12]. Taking the restricted deafferentation model as an example, the representative regions of the transected nerve (median nerve) in the primary somatosensory cortex immediately turn silent to the stimulation of corresponding skin areas, and the unresponsive state commonly lasts for a few hours [23]. Then, the silent cerebral cortex becomes responsive to inputs from the adjacent skin fields in part within several hours to several days whose representative regions in the primary somatosensory cortex are normally adjacent to the transected nerves' [22]. The immediate reactivation within several hours to several days after nerve injury is attributed to the rapid reduction of gamma-aminobutyric acid receptors modulating the fast inhibitory neurotransmission in layer IV of deprived cortical regions, which permits the exhibition of preexisting subthreshold excitatory inputs from adjacent skin fields [24]. The deprived cortical regions continue to undergo complete territorial reactivation within the following 3 to 4 weeks, which is due to the strengthening of preexisting subthreshold inputs, latent correlations, and the persistent reduction of gamma-aminobutyric acid receptors at the binding level after deafferentation [25]. Normally functioning N-methyl-D-aspartate receptors play a decisive role in the initiation of complete territorial reactivation [24, 25]. When nerve transection is combined with the administration of N-methyl-D-aspartate receptor antagonists, 75% of the deprived cortical regions remain unresponsive to peripheral stimulation 4 weeks after deafferentation [24, 25]. Dramatic internal topographic reorganization, characterized by the sharpening of roughly somatotopic receptive fields to more distinct receptive fields, persists in the reactivated cortical regions four weeks later in conjunction with daily use and rehabilitation [22, 23, 26, 27]. At this phase, the refinement of complete territorial reactivation is due to upregulation of aminomethyl phosphonic acid receptors, expression of latent synapses, and axonal sprouting from neighboring cortical regions into the deprived areas [25, 27–30]. On the other hand, the sprouting of cutaneous nerve fibers in the skin does not occur over all the entire time frame [31].

The spatial extent of the reorganized somatosensory cortex is approximately 1-2 mm in the restricted model, and it can reach approximately 10-20 mm in the extensive model [29]. The resection of the dorsal roots of the spinal cord at the C5 level as a representative of extensive deafferentation results in identical reorganization patterns in the CNS. In monkeys, the chin responsive region can invade a deafferented hand cortex at a wide distance of 7 mm [32]. The growth of chin afferents from their normal target, the trigeminal nucleus, into the deprived cuneate nucleus was responsible for the increased width of reorganization [33]. Responses to chin stimulation in the cortical regions originally representing hand innervation could be completely abolished by the inactivation of the cuneate nucleus in the brainstem [32]. In primates, deprived cortical regions can never respond to facial skin stimulation above the chin after resection of the dorsal roots of the spinal cord at the C5 level. This is due to the organizational boundary limitation of new long-projection afferents [32]. In conclusion, large-scale reorganization in the primary somatosensory cortex following extensive deafferentation is mainly due to the growth of new long-projection afferents which occur at the level of the brainstem nuclei and thalamus rather than the plasticity of cortical regions [33–38]. In addition to the growth of long-projection afferents, increased glial activation in the thalamus reflecting the continuous alteration of peripheral afferents was also revealed by positron emission computed tomography in humans which can persist for many years after deafferentation [39].

The transection of motor fibers directly precludes the information outflow of the motor cortex, and the cortical motor regions corresponding to denervated muscles also immediately turn silent [40, 41]. A few hours later, the silent motor cortex shifts its descending projections to the new muscle groups. Electrical stimulation of the deprived motor regions can yield the activity of muscles initially driven by the adjacent motor representations [41–43]. Taking forearm amputation as an example, the shoulder representation in the primary motor cortex rapidly invades the adjacent regions of forearm muscles within a week, presenting with a dramatic increase in cortical size [40]. In primates with forelimb amputation, stimulation of the motor cortex, originally in charge of the motor function of distal muscles, brings about contractions of proximal muscles in the forelimb stump and shoulder [44, 45]. Transcranial magnetic stimulation and positron emission tomography examinations have revealed the enlargement of hand representation with medial shifting into the original cortical face area in humans suffering from a long-term period of facial palsy [46, 47]. This indicates that mere motor deefferentation is a sufficient stimulus for reorganizational changes in the adult human cortex [40, 43, 47]. The rapid shifting of representation within a few hours is supposedly due to an anatomical framework of preexisting, horizontal projections in the primary motor cortex that traverse representation borders rather than the formation of new synaptic contacts or local sprouting [48, 49].

### 2.2. Plasticity of the CNS Involving Peripheral Nerve Injury and Regeneration

Plasticity of the CNS involving peripheral nerve injury corresponds with the physiological processes of nerve injury and regeneration. At first, the representative cortical regions of the injured nerve turn silent; they are soon invaded by adjacent cortical regions that respond to other inputs or yield new muscle activation (reoccupation phase). In humans, increased two-point discrimination ability was observed near the lip, and there was a mislocalization of stimulation of the ulnar side of the fourth finger to the third finger after local anesthetic blockade of the radial and median nerves [50]. Transient anesthetic deafferentation of the radial nerve at the elbow was found to lead to a rapid modulation of the cortical processing of median nerve input and output in humans [51]. Then, there is a phase in which the cortical organization displays the combined effects of nerve injury and regeneration (intermediate phase) [31]. The reactivation of silent regions in the somatosensory cortex commonly emerges in a specific manner, reflecting the sequential proximal to distal sensory nerve reinnervation process [31]. After complete regeneration, cortical topography is reestablished, and preinjury periphery-to-cortex correspondences can be reconnected [52]. Finally, internal topographic reorganization can persist in the somatosensory cortex and is reshaped by rehabilitation or daily usage (internal remodeling phase). However, preinjury cortical topography can never be completely recovered [31]. The restoration of cortical topography differs depending on the type of nerve injury, namely, crush or transection [52]. Nerve transection is more likely to lead to misdirected axonal outgrowth than nerve crush, resulting in altered somatotopic representation [52]. In monkeys with transected and regenerated median nerves, recording sites with abnormally located or multiple cutaneous receptive fields and major topographical changes such as reestablishment digit representations in small discontinuous patches of the cortex were revealed [31]. In contrast, almost normal hand representation, which was in a proximal-to-distal (palmar-to-digital skin) cortex arrangement and continuous manner, recovered and could be observed after crushed median nerve injury [31, 52]. Infant monkeys could attain superior restoration of cortical somatosensory maps and sensory function after median nerve transection compared with adults [53]. Hence, the degree of eventual restoration of normal topography in the deprived somatosensory cortex after peripheral nerve injury depends on the correct axonal outgrowth. With regard to motor nerve lesions, the long-lasting changes at a higher motor cortical level include the shrinkage of corresponding area representing the injured peripheral efferents and the reduction of its excitability, which can be observed even after complete nerve regeneration [54].

In clinical practice, nerve repair or transfer is often performed one or several months after peripheral nerve injury. After peripheral nerve injury and regeneration, Ia afferent information regarding muscle length and dynamics is permanently lost from ventral spinal circuits, which degrades motor performance after complete nerve regeneration [55]. Does the delay in nerve repair cause irreversible structural modifications that have a negative impact on the restoration of normal cortical representation, similar to that in the spinal cord? Unfortunately, few experiments have focused on this issue. We can confirm that the deprived cortical regions can be reactivated to original or new charges even after a long period of deprivation. After four years of denervation, hand and arm representations can return to their original cortical areas 6 months after hand graft surgery [56]. Thus, cortical reactivation cannot be the main barrier for functional recovery in patients with peripheral nerve injury.

## 3. CNS Plasticity after Peripheral Nerve Transfer

Nerve transfer can be considered a special scenario of peripheral nerve injury and regeneration. The physiological processes of nerve regeneration after PNT consist of the following three stages: (1) the transection of healthy donor nerves, (2) the regeneration of donor nerves into the targeted muscles or skin areas (morphological reinnervation), and (3) transformation from morphological reinnervation to functional reinnervation. Functional and structural changes in the CNS after PNT during the first two stages are similar to those involving peripheral nerve injury. The regenerated nerve fibers can be regarded as misdirected axonal outgrowth in consideration of the restoration of normal cortical topography, initially representing the donor nerve. Consequently, the well-organized cortical area of the donor nerve is often transformed into an ill-defined, mosaic-like area in the third stage after PNT [11]. However, the plasticity of the CNS in the third stage plays a paramount role in better functional recovery. This has not yet been fully recognized in clinical practice.

### 3.1. CNS Plasticity Involving Cross Reinnervation of Motor Nerves

#### 3.1.1. The Inherent Anatomical and Physiological Features Contributing to the Neuroplasticity of the Motor Cortex for Excellent Clinical Outcomes of Nerve Transfer

Oberlin's procedure (in which a fascicle of the ulnar nerve innervating the flexor carpi ulnaris is transferred to the musculocutaneous nerve) is a typical example of nerve transfer; it was initially developed and performed in 1994, and it has, over the years, transformed into a first-line procedure for the restoration of elbow flexion due to its ≥90% success rate [57]. Similar excellent results have been achieved for partial ipsilateral C7 transfer and radial-to-axillary nerve transfer [58–60]. The favorable clinical outcomes have been attributed to both short nerve regeneration distance and plasticity of the CNS [12]. Using Oberlin's procedure as an example, the distance between the anastomosis site and the targeted biceps brachii is no more than a few centimeters, which dramatically diminishes the deleterious effects of prolonged denervation [12]. With regard to neuroplasticity, the cortical regions representing the donor and acceptor nerves in the motor cortex are adjacent to each other which may receive partially identical descending corticospinal projections.

**Figure 1:**
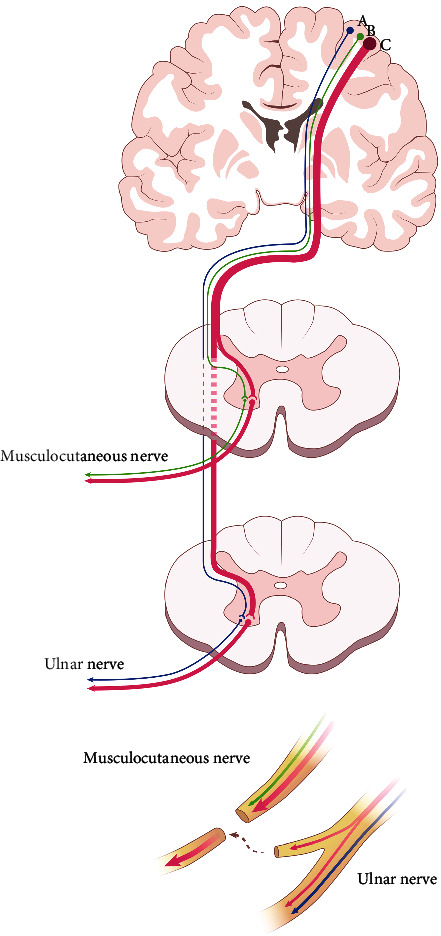
Neuroplasticity of the motor cortex for excellent clinical outcomes of Oberlin's procedure. Corticospinal neurons (corticospinal projection A, B, and C) in the primary motor cortex of layer V projecting into the motor neurons in the spinal cord are the ultimate descending pathways responsible for movement control. A portion of corticospinal projections (corticospinal projection C) can simultaneously connect the ulnar (donor nerve) and the musculocutaneous nerves (acceptor nerve). Hence, the motor command of elbow flexion can quickly be transmitted downward along the common pathway Corticospinal projection C to the ulnar nerve after Oberlin's procedure.

It is well known that the upper, middle, and lower trunks of the brachial plexus send out nerve branches and innervate the muscles of the upper limbs [61]. The corticospinal projections connecting to the three trunks of the brachial plexus are found to be intermingled in the primary motor cortex, secondary motor cortex, primary somatosensory cortex, and secondary somatosensory cortex, and a third of them connect to two trunks [61]. A considerable number of corticospinal neurons innervate both the cervical and lumbar spinal cord [62]. Hence, we can infer that a portion of corticospinal projections initially connects both the ulnar (donor nerve) and the musculocutaneous nerves (acceptor nerve). Detectable reactivation of the cortical area during flexion of the injured elbow in the patients who had undergone Oberlin's operation was similar to that observed in a healthy volunteer [63]. Meanwhile, corticospinal neurons are found to exhibit heterogeneous correlations, with movement which includes silent, indiscriminately active, movement-active, and quiescence-active states [64]. Individual cells can lead to novel associations between corticospinal activity and movement across days [64]. Hence, the two principles of neuroplasticity (Figure 1) that contribute to excellent clinical outcomes in patients undergoing Oberlin's procedure are as follows: (1) the cortical regions representing donor and acceptor nerves are adjacent to each other which receive partial common corticospinal projections from the motor cortex and (2) the dynamic changes in physiological correlations between corticospinal neurons and movements [12, 64].

#### 3.1.2. The Cortical Shifting Phenomenon Contributing to Moderate PNT Results

**Figure 2:**
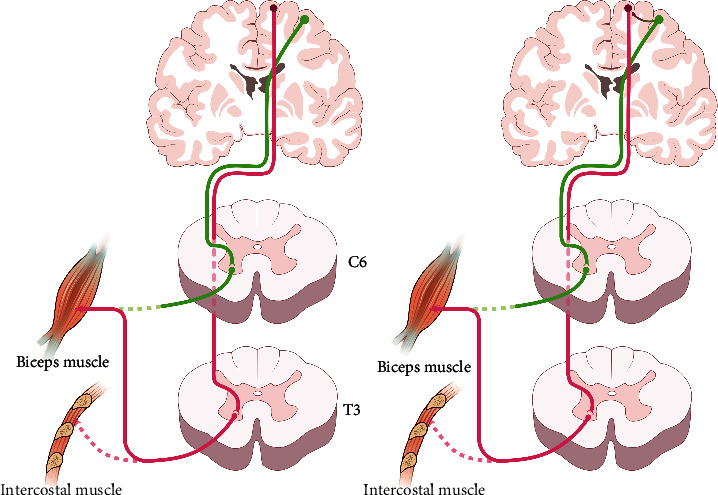
Neuroplasticity after an intercostal-to-musculocutaneous nerve transfer with moderate clinical outcomes. In the early phase of morphological reinnervation (left), biceps contraction is mediated by the original intercostal nerve's primary motor cortex located in the midline. The descending pathway for elbow flexion from the motor cortex to the motor neuron pool of the intercostal nerve T3 is shown in red. Several years later (right), patients begin to contract their biceps independently of respiration. The cortical region representing musculocutaneous nerve delivers the motor control command to biceps brachii via the new relay diaphragm area. The new connection between the 2 cortexes (curved arrow) is reactivated.

Intercostal-to-musculocutaneous nerve transfer performed in patients with complete brachial plexus avulsion, which has an average success rate of 60%-70%, can serve as a typical method for understanding the cortical shifting phenomenon (Figure 2). At the beginning of functional recovery, the contraction of the biceps muscle should be sustained by movements on inspiration or expiration [65]. One to 2 years after intercostal-to-musculocutaneous nerve transfer, elbow flexion can gradually be maintained without the assistance of respiratory movement [65]. Meanwhile, complete independence between the respiratory and elbow flexion movements can never be achieved [65]. The most excitable area of the motor cortex for evoking motor-evoked potential of reinnervated biceps muscle showed a gradual shifting from the cortical map of the intercostal muscles to the arm territory during the same 1-2-year period after intercostal-to-musculocutaneous nerve transfer. This has been termed the cortical shifting phenomenon [65]. Connection of the preexisting cortical network of interneurons in arm representation with intercostal corticospinal neurons is thought to be responsible for this cortical shifting phenomenon [65, 66]. Similarly, patients with complete brachial plexus avulsion, who receive phrenic-to-musculocutaneous nerve transfer for the restoration of elbow flexion, have the same course of clinical recovery and cortical shifting phenomenon of the motor cortex in the third stage as those with intercostal-to-musculocutaneous nerve transfer [67]. The bold fMRI data analyzed using the dynamic causal modeling method indicate that the new neuroplastic connection between the arm and the diaphragm area indeed occurs [66]. A portion of the cortical regions representing phrenic nerves could be gradually separated for the charge of the new elbow flexion function [66, 67]. The connection between the separated and original cortical regions weakens, and the separated cortical regions make new connections with the deprived arm area [66, 67]. Finally, the cortical region representing the arm area delivers the motor control command of elbow flexion to the cervical spinal cord via the new relay diaphragm area [66, 67]. From this perspective, the donor and acceptor nerves must have some horizontal intrinsic connections between the two motor areas, laying the foundation for the cortical shifting phenomenon [12, 66]. The projection distance of the preexisting cortical network of interneurons (horizontal intrinsic connections) may determine the occurrence of the cortical shifting phenomenon [12, 66]. The technique of anastomosis, end-to-side or end-to-end neurorrhaphy, employed during PNT seems to determine the pattern of motor organization [68]. In rodents with intercostal-to-musculocutaneous nerve transfer, the motor representation of biceps muscle was completely reverted to the original biceps area 10 months later after end-to-end transfer [68]. At the same time point, part of the biceps representation remained in the original diaphragm area in the end-to-side group which was manifested as partial cortical shifting [68]. Similarly, both cortical diaphragm and arm areas in patients with the employment of end-to-side neurorrhaphy during phrenic-to-musculocutaneous nerve transfer were activated during elbow flexion [69].

Both Oberlin's procedure and intercostal-to-musculocutaneous nerve transfer have been employed for the restoration of elbow flexion. With regard to Oberlin's procedure, the donor nerve fascicle innervating the flexor carpi ulnaris is initially responsible for both wrist and elbow flexion, which is synergistic with the movement innervated by the musculocutaneous nerve. However, the intercostal nerve, which is used in intercostal-to-musculocutaneous nerve transfer, is a nerve for inspiration/expiration that is completely unrelated to elbow flexion. The difference in movement between the donor and acceptor nerves also is an important factor for determining clinical outcomes [12].

Contralateral C7 nerve transfer was developed for the treatment of patients with brachial plexus avulsion injury for the restoration of shoulder abduction and elbow flexion [70]. In these patients, bold fMRI also showed that the motor cortex representation of the reinnervated upper limb shifts from the ipsilateral to the contralateral hemisphere after long-term remodeling [71]. The injured limb can be moved by stimulating the contralateral motor cortex [72]. The anatomical pathway and mechanism for the contralateral motor cortex to control the movement of the injured forelimb after contralateral C7 nerve transfer occur via the subcortical connectivity [73]. In patients, high level of cerebral glucose metabolism in the corpus callosum was positively correlated with motor recovery of the injured hand 4 years after contralateral C7 nerve transfer [74].

The presence of cortical shifting after ipsilateral and contralateral PNT demonstrates its paramount importance for good motor recovery and voluntary movement control, which may be due to the modifications of the excitatory projections from layer II/III to layer V in the primary motor cortex [75]. Furthermore, the corticospinal and corticostriatal neurons in layer V also received projections from layer II/III to layer V of contralateral cortical areas through the callosum. Cortical shifting in patients with contralateral C7 nerve transfer may also be due to contralateral projections through the callosum.

#### 3.1.3. The Lack of Cortical Shifting Phenomenon Resulting in Unfavorable PNT Results

**Figure 3:**
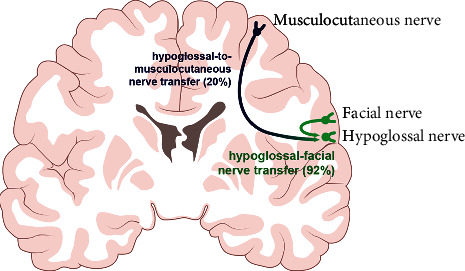
Neuroplasticity responsible for unfavorable clinical results after a hypoglossal-to-musculocutaneous nerve transfer. The distance between the tongue cortical area (hypoglossal nerve) and the arm representation (musculocutaneous nerve) is greater than that between the tongue cortical area (hypoglossal nerve) and the musculus facialis representation (facial nerve). This distance obscures the formation of the new connection to generate the cortical shifting.

Independent voluntary control over reinnervated muscles can never be achieved after hypoglossal-to-musculocutaneous nerve transfer, and involuntary contraction of the biceps muscle can be evoked by talking or chewing [12, 76]. Meanwhile, only 21% of patients achieved M3 or higher elbow flexion strength according to the Medical Research Council's guidelines. In contrast, good results (a 92% success rate) can be expected after unilateral hypoglossal-facial nerve transfer [77–80]. The distance between the tongue cortical area and the arm representation is greater (Figure 3), and this distance may preclude the formation of a new pathway and the germination of cortical shifting in hypoglossal-to-musculocutaneous nerve transfer [76].

#### 3.1.4. The Effects of Antagonistic Movements on Nerve Transfer

Modern concepts of plasticity should also be considered when performing antagonist nerve transfer (the donor and receptor nerves innervating the antagonistic muscles). In patients with proximal median and ulnar nerve injury, transferring the radial nerve branch innervating the extensor carpi radialis brevis to the anterior interosseous nerve can bring about the recovery of full finger and thumb flexion with muscle strength reaching M4 [81, 82]. Pronator quadratus to extensor carpi radialis brevis muscle motor branch nerve transfer can yield a 90% success rate for the reconstruction of wrist extension scored M4 in patients with C5-8 brachial plexus palsy [83–85]. Meanwhile, partial ulnar nerve transfer to the branch of the long head of the triceps has been performed to recover elbow extension, and 90% of patients can achieve M4 or higher elbow extension strength [86–88]. We can come to a conclusion that antagonist nerve transfer in the upper limbs is an effective method. Nevertheless, antagonist nerve transfer does not work in the lower limbs. Tibial branch-to-deep peroneal nerve transfer has been performed for the restoration of ankle dorsiflexion, and only approximately 25% of patients can achieve M3 or greater motor recovery [89]. More studies have to be done before drawing final conclusions on neuroplasticity antagonist nerve transfer [12].

### 3.2. CNS Plasticity Involving Cross Reinnervation of Sensory Nerves

Peripheral nerve injury causes not only motor dysfunction but also loss of sensation [90]. The loss of protective sensation, especially the loss of temperature and pain sense, can lead to secondary physical injuries and can even ultimately compromise the recovery of motor function [90]. For these reasons, restoration of sensation has been emphasized and has been performed in both upper and lower extremities [90–97]. How is the cerebral cortex reorganized after the cross reinnervation of sensory nerves? The proximal ulnar nerve was sutured to the distal radial nerve at the wrist level in a monkey to interpret this issue [98]. The median nerve skin was consistently located in the cortical region responding to ulnar nerve inputs without cortical shifting to the original area after operation, even 2.9 years later [98]. In other words, topographies in the primary somatosensory cortex are relatively stable and their preservation does not depend on peripheral sensory inputs [99]. We should note that this sensory cortex organizational pattern is distinct from that in the motor cortex.

## 4. The New Application of PNT and the Modulation of Neuroplasticity for Better Functional Recovery after PNT

PNT has been newly performed for the restoration of limb function in patients with spinal cord injury, which leads to similar functional improvements in those treated with a tendon transfer [3, 4, 6, 90]. The surgical procedure for performing nerve transfer should be based on the functional level of spinal cord injury and the individual's needs [100]. Meanwhile, contralateral C7 nerve transfer from the nonparalyzed side to the paralyzed side has been creatively performed in patients with chronic cerebral injury to ameliorate the spasticity of the affected upper limb, and satisfactory motor functional improvement has been obtained during the one-year follow-up [101]. This fresh application of the contralateral C7 nerve, which was developed in 1992 by Gu et al., is based on much insight into the fundamental rules of neuroscience implicit in the difference between motor and sensory cortex organization [102]. In these patients, extension movement of the paralyzed wrist was preoperatively correlated with weak activation in the contralateral hemisphere (injured side), which became even more dismal within the 1-year follow-up after contralateral C7 nerve transfer [101]. The newly emerging activation region in the ipsilateral hemisphere generated by extension of the wrist of the paralyzed arm could be revealed by bold fMRI from the 8th month after surgery [101]. In patients with unilateral chronic brain injury, the ipsilateral hemisphere can eventually be responsible for partial control of the motor function of the paralyzed upper limb within one year of follow-up [101].

As maladaptive central plasticity contributes to chronic dysfunction after nerve damage, techniques that reestablish normal central network signaling should improve functional recovery. Motor cortex stimulation can be employed to enhance functional recovery, nerve regeneration, and muscle reinnervation after PNT [103]. Direct stimulation of the motor cortex modulates CNS plasticity for better functional recovery after PNT [12]. Recently, closed-loop vagus nerve stimulation has been shown to improve sensorimotor recovery by enhancing central plasticity, even in the absence of changes to the damaged nerve itself [13, 104, 105]. Rehabilitation, drugs, and electrical stimulation have been commonly employed to improve the nerve regeneration and promote adaptive circuit changes after peripheral nerve injury [106]. Rehabilitative therapies combined with vagus nerve stimulation have emerged as a new trend in targeted plasticity therapy [105, 106]. Meanwhile, the reconstruction of sensory inputs should be emphasized to enhance motor results in clinical practice.

## 5. Conclusion

The keys to successful PNT under the guidance of modern concepts in neuroplasticity are as follows: (1) donor nerves should be properly selected for the targeted muscles; (2) cortical regions representing donor and recipient nerves should be as close to each other as possible; (3) preoperative training of the movements required to activate the nerve transfer should be reinforced; (4) plasticity should be reinforced, especially during the early stages of motor relearning; and (5) well-designed rehabilitation programs with strengthening exercises should be initiated after the observation of initial motor movement [12, 107].
